# Neuromuscular Weakness and Paralysis Produced by Snakebite Envenoming: Mechanisms and Proposed Standards for Clinical Assessment

**DOI:** 10.3390/toxins15010049

**Published:** 2023-01-06

**Authors:** Philip E. Bickler, Michael Abouyannis, Ashish Bhalla, Matthew R. Lewin

**Affiliations:** 1 Center for Exploration and Travel Health, California Academy of Science, San Francisco, CA 94118, USA; 2 Anesthesia and Perioperative Care, University of California at San Francisco, 513 Parnassus Ave, Medical Science Room S-257, San Francisco, CA 94143-0542, USA; 3 Centre for Snakebite Research and Interventions, Liverpool School of Tropical Medicine, Liverpool L3 5QA, UK; 4 Department of Internal Medicine, Postgraduate Institute of Medical Education and Research, Chandigarh 160012, India; 5 Ophirex, Inc., Corte Madera, CA 94925, USA

**Keywords:** snakebite, neurotoxin, anesthesia, paralysis, weakness, presynaptic, postsynaptic, train-of-four, ToF, neuromuscular junction

## Abstract

Respiratory and airway-protective muscle weakness caused by the blockade of neuromuscular transmission is a major cause of early mortality from snakebite envenoming (SBE). Once weakness is manifest, antivenom appears to be of limited effectiveness in improving neuromuscular function. Herein, we review the topic of venom-induced neuromuscular blockade and consider the utility of adopting clinical management methods originally developed for the safe use of neuromuscular blocking agents by anesthesiologists in operating rooms and critical care units. Failure to quantify neuromuscular weakness in SBE is predicted to cause the same significant morbidity that is associated with failure to do so in the context of using a clinical neuromuscular block in surgery and critical care. The quantitative monitoring of a neuromuscular block, and an understanding of its neurophysiological characteristics, enables an objective measurement of weakness that may otherwise be overlooked by traditional clinical examination at the bedside. This is important for the initial assessment and the monitoring of recovery from neurotoxic envenoming. Adopting these methods will also be critical to the conduct of future clinical trials of toxin-inhibiting drugs and antivenoms being tested for the reversal of venom-induced neuromuscular block.

## 1. Respiratory Muscle Paralysis Is a Common Factor in Snakebite Envenoming Lethality

The modernization of treatments for the acute- and long-term effects of snakebite envenoming (SBE) is essential to meet the WHO Roadmap goals for reducing death and disability from snakebite by more than 50% by 2030 [1]. Weakness resulting in the loss of airway protective reflexes and respiratory muscle failure is often the most immediately life-threatening effect of SBE. This lethality is a significant health and economic burden worldwide. In some neurotoxic snakebites, such as those from kraits (genus *Bungarus*), life-threatening paralysis occurs in more than half the patients even though the onset of signs can be delayed by many hours [2,3]. To sustain life when paralysis is developing, airway protection and mechanical ventilation are essential. Depending on the snake species involved, neuromuscular paralysis can co-exist with other clinical manifestations of envenoming, such as local tissue necrosis by elapids (e.g., cobras [4]), organ system failure and venom-induced consumptive coagulopathy (e.g., vipers [5] and some Australasian elapids [6]). Except for major bleeding events, which tend to be rare where treatment is available, other systemic toxicities are rarely as immediately life threatening as neurotoxin-induced respiratory muscle paralysis or airway compromise.

Currently, the tools available for the standardized assessment and severity grading of neurotoxic envenoming are limited, but there is a recent resurgence in the use of the snakebite severity score (SSS) as a clinical tool originally developed for the evaluation of North American crotalid envenoming [7]. Herein, we review the clinical presentation and underlying mechanisms of venom-induced neuromuscular blockade and consider the utility of adopting clinical management methods originally developed for the safe use of neuromuscular blocking agents by anesthesiologists in operating rooms and critical care units. Specifically, we propose that the quantitative monitoring of neuromuscular block and understanding its neurophysiological characteristics may ultimately be key to adopting clear, objective clinical management methods, reducing mortality and meeting the WHO Roadmap goals. In addition, adopting these methods and assessments will be key for future clinical trials of toxin-inhibiting and antibody-based therapeutics.

## 2. Clinical Syndromes of Paralytic Snakebite Envenoming

Descriptions of neurotoxic snakebite are detailed in ancient texts and treatises, including the Bible, and in reference to the travels of Alexander the Great in India [8]. The severity of neuromuscular weakness/paralysis in envenoming ranges from mild to life threatening, depending on the type or quantity of toxin. The two dominant, paralytic toxins are the non-enzymatic α-neurotoxins falling into the three-finger toxin (3FTx) class, found exclusively in elapid venoms, and the enzymatic β-neurotoxins of which the most notable are Type I and Type II secretory phospholipase A2s (sPLA2) found in elapid and viperid venoms. The 3FTx and sPLA2s toxins cause weakness and paralysis by different biochemical mechanisms but both result in interrupted neurotransmission at the neuromuscular junction (NMJ). A venom might contain one or both toxins in varying amounts. For example, many elapids use both α- and β-neurotoxins for prey capture, but viper venom paralytic neurotoxins are exclusively from the β class. Two other distinct types of neurotoxins are the fasciculins and dendrotoxins, both of which are present in the venom of the *Dendroaspis* species while some rattlesnakes, for instance, induce ataxia through induction of myokymia via poorly understood peptide toxins alone or interacting with sPLA2 [9].

Neurotoxic envenoming is most often described to manifest as a “descending paralysis” progressing from small to large muscle groups. A typical descending paralysis progresses affecting smaller muscle groups and may present as ptosis secondary to weakness of the levator palpebrae as the most frequent initial sign, followed by ophthalmoplegia and then diplopia. As in myasthenia gravis, ocular muscles seem particularly susceptible to weakness [10]. Facial nerve weakness is usually then seen, followed by speech slurring and weakness of the masseter muscles. Next, the neck and bulbar muscles are affected, which may manifest as dysphagia, dysarthria, drooling, and airway compromise. Failure of the intrinsic breathing muscles (intercostals and diaphragm) causes reduced tidal volume, often requiring ventilatory support. Paralysis will eventually involve the limb muscles [10,11]. Gupta et al. [12] described a range of neuroparalytic signs in 37 patients envenomed by elapids. Ptosis was found in 22, ophthalmoplegia and pharyngeal paralysis in 12, facial paralysis in 9, respiratory paralysis in 8, flaccid limb paralysis in 16, and “lock jaw” in 1. Other reports also emphasize the common feature of central weakness (i.e., cranial nerves and axial muscles, more so than appendicular muscles), especially as an early sign, and one that may predict progression to respiratory compromise. Descriptions of the severity of weakness, ranging from mild involvement of the eyelids and facial muscles to fatal paralysis of bulbar and respiratory muscles, have been reported in other publications [2,6,13,14,15,16]. In extreme cases, complete neuromuscular paralysis involving all skeletal muscles of the body occurs [16]. While Silva observed considerable between-patient variation, ophthalmoplegia, facial weakness, and ptosis were the earliest observed neuromuscular effects, although respiratory muscle paralysis was the initial sign observed in patients who had a delayed presentation to health services. This pattern of central weakness, including respiratory paralysis, was typically seen before lower limb weakness manifested. The persistence of weakness typically follows the same pattern, with ptosis, diplopia, and ophthalmoplegia continuing for longer periods of time than limb weakness such that it appears that some muscle groups are more susceptible to β-neurotoxins or show effects earlier in the paralytic syndrome and when severe may result in long ventilator times despite optimization of ventilator settings [17].

As a general rule, the physical and chemical characteristics of protein and peptide-based toxins prevent them from crossing the blood–brain barrier unless there is capillary leak or hemorrhage. Nevertheless, neuromuscular weakness can have central nervous system (CNS) effects that are secondary to hypoxia and hypercarbia. This includes drowsiness, stupor, and coma. Clinicians caring for patients paralyzed by envenoming must be aware that patients are often not comatose; cerebrocortical function may be completely intact, with preserved pain sensation, auditory processing, and formation of memory. This may be particularly true of patients intubated and ventilated, who are not suffering from hypoxia or hypercarbia-induced CNS depression. The appropriate use of sedative/hypnotic medications such as benzodiazepines and propofol infusions, as well as pain relief with opioids, non-steroidal anti-inflammatory drugs, or ketamine is important. Patients who have been paralyzed and recover later relate terrifying stories of being too weak to communicate while suffering from pain and severe anxiety. Post-traumatic stress disorder after SBE is not uncommon and represents a significant cause of morbidity [18]. Conversely, neurological evaluations taking place after removal of sedation should consider the anticipated duration of sedation and its removal to perform meaningful neurological examinations.

## 3. Pharmacology of Paralytic Snake Venom and Neuromuscular Blocking Drugs

The tools currently available for the standardized assessment and severity grading of neurotoxic envenoming are limited and subjective. To develop an objective clinical assessment tool, we propose that there is utility in examining methods originally developed for the safe use of neuromuscular blocking agents that are mature in the field of anesthesiology and perioperative care. Numerous similarities exist in the mechanisms of pharmacologic action between paralytic snake venom components and clinically used neuromuscular relaxant drugs. It is important to consider two categories of clinically relevant drugs: those that depolarize the postsynaptic membrane while causing weakness or paralysis and those that do not. This division groups the compounds by their clinical effects and the mechanism of action on the postsynaptic membrane. This categorization is summarized for venom components and neuromuscular blocking drugs in Table 1.

### 3.1. Depolarizing Toxins and Drugs

#### 3.1.1. β-Toxins (Venom sPLA2 Neurotoxicity)

**Figure 1:**
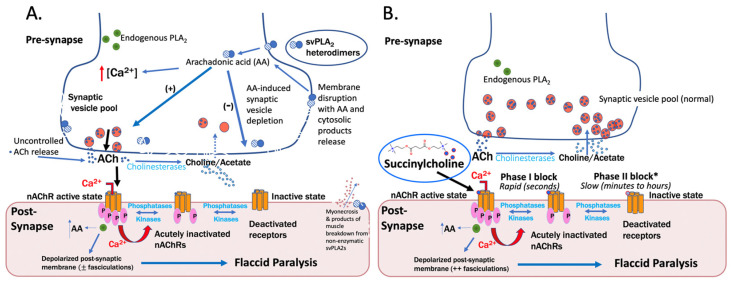
Similarities and differences in mechanisms and effects of depolarizing neuromuscular block produced by (**A**) β-neurotoxins and (**B**) Succinylcholine. β-toxins and succinylcholine share the postsynaptic effects of depolarization and flaccid paralysis but diverge in that succinylcholine acts exclusively on the postsynapse while snake venom sPLA2 (svPLA2) classified as β-neurotoxins toxins produce effects directly on both the presynapse with postsynaptic sequelae resulting in paralysis and muscle membrane damage oftentimes from non-enzymatic svPLA2s. Postsynaptically, both classes of agents cause hyperactivation of nicotinic receptors (nAChRs), via uncontrolled acetylcholine (Ach) release, indirectly in the case of (**A**) β-neurotoxins because of uncontrolled release of Ach and directly in the case of (**B**) succinylcholine by persistent opening of the nAChR. Because activated nAChRs rapidly desensitize, profound but brief paralysis occurs with rapidly metabolized succinylcholine and prolonged weakness with sPLA2 toxins that also damage presynapse membranes. * Very rarely, succinylcholine can case Phase II block related to disease or repeated administration. Disruption of the presynaptic membrane and other inflammatory events may result in arachidonic acid-induced reductions in synaptic vesical regeneration with consequences for repopulation and further reduction in strength of synaptic transmission.

β-toxins produce paralysis by more complex and less well-understood mechanisms than other toxins, such as curare or three-finger toxins. A summary of the pre- and postsynaptic effects of β-toxins is presented in Figure 1a. β-toxins are multimeric and their action is multifactorial, including a primary presynaptic effect of synaptic vesicle release and depletion, as well as postsynaptic nicotinic receptor desensitization and inactivation. This pathophysiology has, to date, been found resistant to antivenom-treatment, and recovery seems to depend on the natural restoration of nerve terminal integrity as shown from experimental studies using presynaptic toxins isolated from krait and viper venoms [19,20,21,22]. Importantly, if the β-neurotoxin-like depolarizing block is maintained, nicotinic receptors become deactivated and may become unresponsive to reversal of block by anticholinesterases such as neostigmine.

#### 3.1.2. Pharmacologically Depolarizing Neuromuscular Blockers

Currently, succinylcholine is the only depolarizing neuromuscular blocker used clinically. The short and long-term effects of succinylcholine are shown in Figure 1b. Depolarizing agents such as succinylcholine have a bi-phasic effect on nicotinic receptors, much like nicotine mimetics: they initially hyperactivate nicotinic receptors and depolarize the postsynaptic membrane, and secondarily cause long-lasting receptor desensitization and inactivation. Inactivation occurs by receptor dephosphorylation [23].

The initial activation produces what is called a phase I depolarizing block, and the deactivation is termed a phase II block. Phase I and phase II blocks have distinctly different durations of clinical effect and produce distinctly different patterns of response to neuromuscular function monitoring (described below). The processes by which succinylcholine produce neuromuscular block are shown in Figure 1b, below.

The phase I depolarizing block produces visibly disorganized muscle contractions, a phenomenon known as fasciculation. Fasciculations may be barely noticed in the elderly but can be quite dramatic in young muscular patients. The contractions produced can lead to myalgias. Historically, since fasciculations can be blocked by non-depolarizing agents, small doses of curare were given before succinylcholine. However, the non-depolarizing effect will antagonize the depolarization that produces the paralysis that is the desired effects of succinylcholine, so it is not used when very rapid paralysis is required, such as in the emergency management of a difficult airway or when regurgitation and aspiration of stomach contents might occur. The combination of succinylcholine and NDNMB administration may effectively mimic elapid envenoming where there are mixed α- and β-toxin effects.

#### 3.1.3. Reversal of Succinylcholine Effects

The reversal of the block from depolarizing muscle relaxants (succinylcholine) occurs by the process of rapid drug metabolism and spontaneous resolution of nicotinic receptor desensitization and inactivation. Normally, plasma butyrylcholinesterases rapidly metabolize succinylcholine, reducing its duration of action to less than five minutes. However, if succinylcholine is present for a prolonged period (as when given by constant infusion or in a patient with genetic cholinesterase deficiency), receptors can remain deactivated. Therefore, patients with genetic defects resulting in the lack of plasma cholinesterase are susceptible to developing long-duration and difficult-to-reverse neuromuscular block if they are given succinylcholine. Similarly, succinylcholine can exacerbate myasthenia gravis and result in prolonged weakness. Cholinesterase inhibitors for the reversal of phase II block are contraindicated when the neuromuscular block has been caused by depolarizing agents (e.g., succinylcholine or similar drugs) because additional acetylcholine in the NMJ, resulting from cholinesterase inhibition, increases nicotinic receptor desensitization, preventing reversal of the block. If reversal is attempted under these conditions, the duration of the paralysis can be prolonged because receptors are converted from a desensitized to an inactivated state. When a phase II block from succinylcholine has occurred, one must simply wait until the block resolves spontaneously, which may require more than an hour.

#### 3.1.4. Fasciculins

Fasciculins comprise an interesting and lesser-known group of three-finger toxins that produce neuromuscular dysfunction. These toxins cause fasciculations by inhibition of acetylcholinesterase and uncontrolled accumulation of acetylcholine at the NMJ, an entirely different mechanism from the nicotinic receptor antagonism of other 3FTxs (e.g., rattlesnakes). The ensuing exuberant activation of nicotinic receptors causes muscle depolarization, fasciculations, and weakness. The effects of fasciculins are similar to the phase I and II neuromuscular blocks caused by succinylcholine [24,25].

#### 3.1.5. Nerve Agents

Nerve agents produce neuromuscular weakness by inhibiting acetylcholinesterase, like fasciculins. Acetylcholinesterase inhibition causes acetylcholine accumulation and nicotinic receptor hyperstimulation, leading to receptor desensitization and inactivation. The inactivation of nicotinic receptors is analogous to receptor inactivation in succinylcholine-induced phase II block (see also Figure 1b, above). The nicotinic receptor overactivation that precedes paralysis causes visible fasciculations in major muscle groups. Cholinergic over-activation also leads to other signs of nerve agent toxicity, including bradycardia, diarrhea, and the excessive saliva production seen in mamba envenoming in particular.

### 3.2. Non-Depolarizing Toxins and Drugs

#### 3.2.1. Three-Finger Toxins (3FTxs, α-Toxins)

The 3FTxs superfamily contains three β-chain loops that protrude as fingers. These non-enzymatic toxins act postsynaptically to competitively inhibit nicotinic receptors, similar to curare and curare-like drugs, by binding at two agonist binding sites on the adult muscle type of nicotinic receptors (α1-δ and α1-ε interface of (α1)_2_βδε) leading to nerve block [26]. Two subclasses of the 3FTxs family are the short chain α-neurotoxins, with 61–62 amino acids and 4 conserved disulfide bonds, and the long-chain α-neurotoxins, with 66–75 amino acids and 5 disulfide bonds [27]. Proteomic and clinical data suggest that paralysis in human snakebite is primarily associated with long-chain α-neurotoxins, such as those from cobras [27]. Snake venom neurotoxins competitively bind with high affinity and poor reversibility, blocking neuromuscular transmission [28,29] and posing a challenge for therapeutics. Because these toxins do not depolarize the postsynaptic terminal, they do not cause fasciculations.

#### 3.2.2. Non-Depolarizing Muscle Relaxants

Non-depolarizing neuromuscular blockers (NDNMBs) are a drug category of clinically relevant compounds with curare-like actions that competitively antagonize postsynaptic nicotinic acetylcholine receptors. NDNMBs never cause fasciculations, either during initial effect or recovery. The competitive effects of NDNMBs at nicotinic receptors means that their actions can be reversed with cholinesterase inhibitors. Steroid-based neuromuscular blocking drugs were introduced in the 1960s and short- to intermediate-duration drugs are now ubiquitous in anesthesiology and critical care. Synthetic, steroid-derived drugs include pancuronium, atracurium, mivacurium, vecuronium, and rocuronium. These drugs have completely displaced curare from clinical use, although small-dose d-tubocurarine use persisted through the 1990s for use in preventing fasciculations from succinylcholine.

#### 3.2.3. Reversal of Non-Depolarizing Neuromuscular Drug Effects

Neostigmine, edrophonium, pyridostigmine, and similar agents have been used since the 1950s to reverse the neuromuscular block produced by curare-like drugs and since the early 1930s to reverse weakness from myasthenia gravis as first described by Mary Broadfoot Walker in 1935 [28]. The basis for reversal is the inhibition of cholinesterase at the NMJ, which results in increased acetylcholine concentrations. The increased acetylcholine displaces competitive antagonists of nicotinic receptors, restoring NMJ function. The effectiveness of this reversal is dependent on several factors. First, the degree of binding of the nicotinic blocker drug must be at a level where its blocking effects can be competitively antagonized, i.e., cholinesterase inhibitors can be ineffective or partially effective when the concentration of competitive neuromuscular blocking drug is high. Clinically, this is well established and used by clinicians; for reversal of the block, the neuromuscular blocking drug levels must have dissipated sufficiently for cholinesterase inhibition to restore NMJ function. A second factor is the presence of other drugs or conditions that may augment the depth of neuromuscular block, such as the presence of halogenated anesthetic agents, renal impairment that reduces drug clearance, and acidemia. Physiological changes that may impair reversal of neuromuscular block include CO_2_ retention-induced respiratory acidosis, metabolic acidosis, other medications that potentiate neuromuscular block, and the inappropriate use of cholinesterase inhibitors.

An entirely different strategy for reversing neuromuscular block involves the use of drugs that non-covalently bind steroidal neuromuscular blockers. Sugammadex is the first drug in this class. Sugammadex is a crown ether that has a high affinity for the steroidal drug rocuronium. Reversal of rocuronium neuromuscular block with Sugammadex can be very rapid and complete [29].

#### 3.2.4. Botulinum Toxin

Like β-neurotoxins, Botulinum toxin (BTx) is a multi-subunit toxin that causes neuromuscular transmission blockade by interfering with arachidonic acid metabolism and vesicular release [30]. BTx blocks neuromuscular transmission by cleaving the SNAP-25 protein that is part of the SNARE protein complex/vesicle docking complex in the presynaptic terminal [30]. Because BTx decreases neuromuscular transmission presynaptically, it does not cause fasciculations.

## 4. Reversal of Venom-Induced Weakness and Paralysis

A fundamental difficulty in interpreting studies and case series involving treatments for paralytic envenoming is that standard measures of neuromuscular weakness have not been used. Quantitative assessment of neuromuscular weakness, such as train-of-four monitoring, has been virtually non-existent in studies of antivenom efficacy [10].

### 4.1. Neostigmine and Other Anti-Cholinesterase Drugs

The clinical utility of cholinesterase inhibitors for the reversal of paralysis in SBE remains controversial even fifty years after the first description of its use by Banerjee in 1972 [10,31]. Some clinical reports and experimental studies provide support for at least partial cholinesterase-inhibitor reversal of neuromuscular blockade by α-bungarotoxin and other three-finger toxin venom components [10]. The successful reversal of α-toxins could be considered analogous to the reversal of NDNMBs used in surgery and critical care. There are several reports of the benefits of atropine/neostigmine in paralytic envenoming from elapid bites. Glycopyrrolate is also commonly co-formulated with neostigmine and used in the treatment of snakebite in India. For example, Gupta and colleagues treated 37 victims of elapid envenoming with a combination of antivenom and neostigmine-atropine, with improvement in 25 of 37 cases [10,12]. Watt et. al., and Gatineau also described leading successful studies of reversal of α-toxin venom effects with neostigmine in patients bitten by Philippine cobras (*Naja naja philippinensis*) [32,33].

In contrast to α-toxins, reports have described that the reversal of β-toxin-mediated paralysis does *not* occur with neostigmine [10,34]. This is thought to be due to depletion of synaptic vesicles caused by phospholipase activity at the presynaptic terminal. As noted above, the uncontrolled release of acetylcholine will contribute to another deficit in synaptic transmission—nicotinic receptor inactivation. There is also evidence that β-toxins cause physical changes in the synapse [21,22].

Because fasciculations indicate depolarization of the muscle membrane from excessive and uncontrolled acetylcholine release, cholinesterase inhibitors such as neostigmine should be used with great caution or avoided when β-toxin effects are the primary feature of the envenoming snake or if fasciculin-containing venom is suspected [10,24,34,35].

### 4.2. Antivenom for the Treatment of Weakness and Paralysis

A recent review of the effects of standard-of-care treatment for snakebite envenoming, antivenom, reveals no randomized placebo-controlled trials of antivenom effectiveness in reversing paralysis [11]. As well, according to this review by Silva and colleagues, all the randomized trials, comparative studies, and cohort studies had deficiencies either in the case definition, study design, small sample size, or objectivity of the measures of paralysis [11]. Evidence of efficacy from studies of α-toxin-induced paralysis (non-depolarizing) suggests that antivenom may reverse early signs of neurotoxicity, but placebo-controlled studies with objective assessment of weakness and paralysis are required [11,36,37,38,39,40].

In contrast, studies examining paralysis caused by β-neurotoxins (depolarizing, e.g., kraits, taipans) suggest that antivenom does not reverse or even improve established neurotoxicity, although early administration appears to prevent neurotoxicity and might be associated with a decrease in severity for some snakes, mainly taipans [10]. For example, in 245 victims of confirmed Russell’s viper envenoming, antivenom treatment was ineffective in improving neurological symptoms and did not show significant improvement by single fiber electromyography (sfEMG) [41]. The underlying resistance to antivenom may be due to physical damage to the presynaptic terminal, which appears to occur within hours of envenoming [19], but to our knowledge, these alterations in synaptic ultrastructure have not been observed in human biopsy or autopsy studies of victims of snakebite envenoming. Other possible explanations for antivenom resistance are that antivenom molecules cannot effectively overcome venom sPLA2 binding or, perhaps they are unable to reach synapses because of their size and charge, or that sPLA2 is internalized into the presynapse, and is therefore inaccessible to neutralization [22,37]. As such, small molecule inhibitors of venom sPLA2 are a promising option to treat antivenom-resistant paralysis, but these types of direct inhibitors of non-enzymatic neurotoxins such as 3FTx have yet to be developed [22,42,43,44,45].

Future research should include carefully controlled studies investigating whether the early administration of antivenom will prevent neurotoxicity by both depolarizing and non-depolarizing toxins, and whether antivenom can reverse α-toxin-induced paralysis. This is particularly important for venoms from the main cobra genera (*Naja* and *Ophiophagus*) for which there is great venom diversity by geographic location and, therefore, wildly differing neutralization potential of polyvalent antivenoms [46]. Controlled studies will require better case definition to cleanly separate neuromuscular paralysis caused by depolarizing vs. non-depolarizing toxins and mixed-neurotoxin envenoming syndromes with varying contributions from pre- and postsynaptic actors. It would be additionally helpful for better case definition to be achieved by using venom-specific assays to confirm envenoming [11] and by utilizing the improved objective measures of both clinical and neurophysiological neurotoxicity described below [11].

## 5. Tools for Clinical Assessment of Neuromuscular Function in Snakebite

At present, there are limited tools available for the standardized assessment and severity grading of neurotoxic envenoming. The original snakebite severity score (SSS), although validated as a graded assessment scale in pit viper envenoming and adopted by some investigators outside North America [7], requires significant adaptation before it can be reliably used to assess venom-induced paralysis with sensitivity to anything but profound changes in clinical condition. While the original SSS includes weakness, it neglects other key findings that are observable and/or measurable by clinical examination or instrumental testing. This means that the neurological subscore of the SSS may be of limited utility to address specific questions of neurotoxicity commonly seen outside of North America.

Most recently, the SSS was modified by clinical investigators to include a renal subscore and a modestly updated bedside neurological examination that has been accepted for evaluation by regulatory authorities in the US and India (NCT04996264). Unfortunately, current snakebite clinical management guidelines do not provide a sensitive tool for grading neurotoxicity, in particular for sedated, intubated patients. For treatment, the guidelines are primarily focused on the decision of when and how frequently to administer antivenom, which is generally binary depending on whether any feature of neurotoxicity (or other major toxicity) is present [47]. For example, in India, the current national protocol for neurotoxic envenoming is to start with 10 vials of polyvalent antivenom (ASV), wait one hour, and if the patient has not improved based on the “5 Ds and 2Ps”—dyspnea, dysphonia, dysarthria, diplopia, dysphagia and ptosis, paralysis [48]—then administer another 10 vials for a total of 20 vials and then rely on supportive measures to most safely manage weakness.

Modernizing the SSS and developing a more detailed assessment tool specific to the neurotoxic syndromes in snakebite may not be mutually exclusive exercises and should have complementary utility. A detailed assessment tool would facilitate documentation of the development and recovery of neuromuscular function, as well as provide a quantitative basis for assessing new and older therapeutic platforms for this heterogeneous disease. Based on relatively uniform clinical descriptions of the neuroparalytic syndromes associated with snakebite envenoming, it is reasonable to imagine an expanded weakness severity scale. Such a scale would need to have components not entirely dependent upon specialized equipment or laboratory assessments. Patients on ventilator support would need to be evaluated based on additional parameters that effectively disambiguate clinically induced weakness and conveniences that might interfere with evaluation (e.g., delayed extubation because of a lack of nighttime staff and backup support). Possible measures for the systematic assessment of toxin-induced weakness are shown in Table 2.

### 5.1. Hypothetical Snakebite Weakness and Paralysis Scoring

A detailed assessment tool must address four key aspects of the clinical management of snakebite envenoming:

1. Clinical and quantitative protocols for assessment of venom-induced neuromuscular weakness and paralysis;
2. Optimization of airway management and mechanical ventilation;
3. Monitoring of adequacy of breathing and gas exchange;
4. Understanding utility and limitations of drugs in reversal of weakness and paralysis.

We propose the following hypothetical clinical tool to grade SBE induced weakness and paralysis. Following community discussion and consensus, our proposed four-point grading scale could be incorporated into the SSS as the neuromuscular function component.

Grade 0 No weakness.
Grade 1 Mild cranial nerve deficit (e.g., ptosis) and no bulbar weakness; able to lift limbs and neck against gravity, ambulatory without assistance.
Grade 2 Unable to lift limbs/neck against gravity, difficulty swallowing, or inability to ambulate independently.
Grade 3 Severe weakness causing reduced ventilatory function, but spontaneous respiration is preserved.
Grade 4 Complete paralysis; reflexes absent; ventilator dependent.

The distinction between grades would be based on clinically relevant deterioration or improvement that can be conveniently scored as a patient worsens or improves. For example, Grade 2 involves cranial nerve dysfunction, but there may be preserved intercostal/diaphragm muscle function, and anticipation of the need for airway protection. With Grade 3, the primary muscles of respiration are weak, with hypoventilation even when patient is protected from airway obstruction with an airway device or intervention. Grade 4 requires mechanical ventilation for survival and can be downgraded to a Grade 3 as spontaneous respiration returns. For sedated and intubated patients evolving between Grade 2 and Grade 4, the monitoring of neuromuscular weakness can at least partially overcome the limitations of clinical examination obscured by sedative hypnotics as described below. Ideally and when available, quantitative neuromuscular monitoring could help distinguish between weakness states.

### 5.2. Monitoring Neuromuscular Weakness and Paralysis

#### 5.2.1. Quantitative Neuromonitoring

Studies show that the clinical assessment of neuromuscular block can be inaccurate, even when performed by very experienced clinicians. Crude assessments of neuromuscular function can be influenced by many factors, for example, inability to follow instructions or pre-existing physical disability. Patients who have received neuromuscular blocking drugs and are clinically judged to have no significant residual weakness often do have clinically significant residual weakness, to an extent that places them at risk for complications including pneumonia, aspiration, and airway obstruction after extubation [49,50,51]. In addition, it is important to recognize that various underlying physiological conditions can influence the degree of neuromuscular block. Acidemia, secondary to CO_2_ retention, increases weakness. As well, sedative medications such as propofol can increase neuromuscular block. Renal failure prolongs the half-life of neuromuscular blocking drugs, but its effects on venom clearance have not been well studied. In critically ill patients, aminoglycoside antibiotics such as gentamycin can adversely affect neuromuscular function [52] and drugs of this class should be avoided, if possible, in patients being treated for snake venom paralysis. Therefore, the hypothetical scoring system proposed above would ideally be complemented by additional quantitative neuromonitoring methods to assess both neuromuscular block and degree of recovery. Validating and adopting examination standards in neurotoxic envenoming is critical to avert the iatrogenic complications of residual weakness and imperative to improve the quality of clinical trials of snakebite treatments.

#### 5.2.2. Types of Neuromuscular Monitoring

There are two main types of neuromuscular monitoring: acceleromyography where muscle action is measured as the mechanical output of muscle, and electromyography (EMG), where muscle strength is measured based on the electromyogram of a muscle stimulated by a nerve. A recent paper showed that the EMG technique might be preferable in detecting block, but it is also invasive and could be less desirable for patients, families and other stakeholders otherwise encouraged to enroll in clinical studies [53,54].

**Figure 2:**
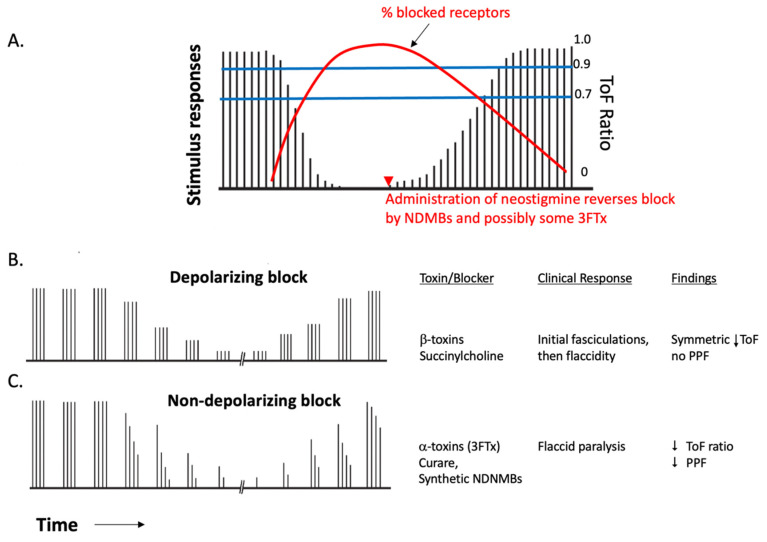
Neuromuscular responses to nerve stimulation with non-depolarizing and depolarizing blocker administration and recovery. Responses to “train-of-four” nerve stimulation are shown as vertical lines. (**A**) Pattern of evoked muscle responses to twitch stimulation after administration of a non-depolarizing neuromuscular blocking drug (NDNMB), followed by antagonism with neostigmine(▼), hastens the rate of recovery, if the twitch has already started to increase. (**B**) Pattern of evoked muscle responses to twitch stimulation after administration of succinylcholine. (**C**) ToF monitoring of onset and recovery from neuromuscular block produced by a NDNMB. Neostigmine can be utilized for reversal of NDNMBs such as naturally occurring curare or synthetics such as pancuronium, vecuronium or rocuronium.

Quantitative neuromonitoring involves the controlled stimulation of a motor nerve and observation and/or quantitative measurement of the muscular response. Clinically, this is typically carried out with four equally spaced stimuli, typically at 60 mA, 1.0 Hz, pulse width 10 ms. A typical pattern of stimulation and response is shown in Figure 2 (Below). The change in magnitude of the response over the four stimuli is termed the train-of-four ratio or “ToF ratio” and relates to the percentage of postjunctional nicotinic receptors that are bound to the competitive blocker [53,54]. NDNMBs are competitive antagonists of acetylcholine at nicotinic receptors; therefore, a burst of stimulation produces a larger response to the first impulse, but because the amount of acetylcholine released next is smaller than the first, the response decays. In contrast, a depolarizing agent such as succinylcholine causes desensitization and then deactivation of the nicotinic receptors, reducing the number of active receptors. A train of four stimulation shows equal depression of all four stimuli. Therefore, ToF could be used to differentiate between paralytic symptoms caused by depolarizing and non-depolarizing toxins.

For clinical trials, there is an urgent need to establish reproducible standardized outcome measures [55]. Although various clinical trials and retrospective studies have reported on the proportion of patients needing assisted ventilation [10,17,40,56,57] or the duration of assisted ventilation [17,56], reproducible definitions have not been used. A core outcome set, developed by critical care clinicians, outlines clear criteria for clinical trials of treatments that modify the need for mechanical ventilation [55,58]. Although determining the onset time of intubation or ventilation is straightforward, defining the point when assisted ventilation has ceased is not as straightforward because there are both clinical and non-clinical reasons for the timing of weaning and documentation needs to be fastidious not just for ventilator settings, but also the timing and doses of concomitant medications.

Most patients recovering from a critical illness are gradually weaned from mechanical ventilation and may transition to non-invasive ventilation before all ventilatory support is removed. Furthermore, patients may initially tolerate a period without assisted ventilation before deteriorating and requiring a temporary period of increased ventilatory support. To overcome this, clinical trials can adopt a definition of “first successful unassisted breathing”, which is defined as continuing to breath without assistance for a period longer than 48 h [55,58]. This definition has recently been incorporated into a proposed core outcome set developed for snakebite clinical trials. Although trial outcome measures based on the need for intubation or mechanical ventilation are highly clinically relevant, more sensitive surrogate measures are needed, particularly to support clinical trials with a smaller sample size. 

In the above-proposed hypothetical snakebite weakness and paralysis scoring system, or adaptations of this tool, may provide a more sensitive outcome measure than solely relying on measures of reliance on ventilatory support. A similar tool has been successful in clinical trials in Guillain–Barré syndrome, which, like neurotoxic envenoming, can cause an acute flaccid paralysis. 

The “GBS disability score” at 4 weeks after randomization is an established primary outcome measure [59]. This scoring system records functional ability from 1 to 7, as follows:

1 Healthy;
2 Minor symptoms of neuropathy but capable of manual work;
3 Able to walk without support of a stick but incapable of manual work;
4 Able to walk with a stick;
5 Confined to a bed or chair;
6 Requiring assisted ventilation; or
7 Dead.

Outcome measures based on physical function such as these are desirable as they are sensitive, patient centered, and clinically relevant [55].

EMG studies also represent a potentially valuable outcome measure for snakebite clinical trials. Only two snakebite randomized controlled trials have included electromyography as an outcome measure. In contrast to the study by Silva and colleagues using sfEMG [42], in the trial by Trevett et al., 50 participants with *Oxyuranus scutellatus canni* envenoming in Papua New Guinea were randomized to edrophonium, diaminopyridine, or placebo, in conjunction with atropine [60]. EMG was used, with stimulation of the ulnar nerve and measurement of the compound muscle action potential (CMAP) at the abductor digiti minimi. The assessment of CMAP identified a significant difference between the intervention and placebo arms of the trial, although this translated into a limited clinical effect. In the trial by Watt et al., 10 participants envenomed by *Naja naja philippinensis* were randomized to receive atropine and edrophonium, or placebo [32]. However, EMG was only performed for two participants and the results were incompletely reported. EMG may be a valuable outcome measure for snakebite clinical trials, with good clinical correlation suggested by observational studies, but ToF might be the more desirable approach for its non-invasive attributes and widespread use in anesthesia monitoring [16,61].

## 6. Conclusions

Standard clinical and quantitative neuromuscular neuromonitoring are needed to improve snake bite care. We advocate a combination of clinical assessments and quantitative neuromuscular monitoring with nerve stimulation. The specific modalities of neuromuscular stimulation include ToF and paired pulse facilitation. Types of monitors, sites for assessment (ulnar nerve, masseter, or facial nerve), sensitivity, and specificity for different sites with depolarizing vs. non-depolarizing agents need to be clarified. The development of these tools may become essential for decreasing mortality and morbidity following snakebite and are desirable as well as likely necessary for the evaluation of therapeutics in future clinical trials.

## Figures and Tables

**Table 1:** Comparison of muscle relaxants, venom toxins, their pharmacological effects and reversal.

**Depolarizing Neuromuscular Blockade**			
**Drug/Venom type**	**Succinylcholine**	**β-toxin (PLA2)**	**Fasciculins**
**Nicotinic receptor** **(nAChR) effects**	Hyperactivation followed by desensitization and inactivation	Hyperactivation followed by desensitization and inactivation	Hyperactivation caused by AchE inhibition
**Post synapse/** **Muscle**	Depolarization, fasciculations	Depolarization, fasciculations	Depolarization
**Effect duration**	Short, may progress to long	Long	Long
**Fasciculation**	Yes	Maybe	Yes
**Reversal with** **neostigmine?**	No, may increase block	No, may increase block	May exacerbate block
**Non-Depolarizing Neuromuscular Blockade**			
**Drug/Venom type**	**Nondepolarizing neuromuscular blockers (NDNMB)** (e.g., curare, rocuronium, vecuronium)	**α- or “3FTxs”** (e.g., α-bungarotoxin)	**Botulinum toxin**
**Presynaptic effects**	None	None	Vesicle depletion
**Nicotinic receptor (nAChR) effects**	High-affinity competitive blocker	High-affinity competitive blocker	none
**Postsynaptic muscle membrane**	none	None	none
**Effect duration**	Long, drug dependent	Long	long
**Fasciculation**	No	No	Rare
**Reversal with Neostigmine?**	Yes	Likely venom dependent	Yes

Abbreviations: NDNMB: non-depolarizing neuromuscular blocking drug, AchE: acetylcholinesterase. Examples of α-toxins are α-bungarotoxin, cobratoxin. Clinically used neuromuscular blocking drugs include rocuronium, vecuronium, pancuronium. Succinylcholine is rapidly metabolized by plasma butyrylcholinesterases and block typically lasts less than five minutes, but in rare instances may cause Phase II block as with repeated doses of succinylcholine can cause prolonged receptor desensitization and inactivation with long-acting paralysis. Fasciculins may produce long-lasting fasciculations. It is uncertain how often β-toxins cause fasciculation that might precede longer-acting presynaptic block. Rate of reversal depends on type, degree, and duration of blockade.

**Table 2:** Tools for evaluation of neuromuscular deficit in setting of snakebite.

	Clinical Exam	Example Assessments	Laboratory Findings
Cranial Nerve Palsies	Ptosis	Photographs	-
	Decreased visual acuity	Vision chart	-
	Tongue protrusion weakness	Clinical assessment	-
	Unable to support head for 5 s	Time to loss of unaided support when sitting	-
	Upper airway obstruction	Masseter ToF, chest movement monitors (e.g., Respitrace™), capnography	Hypercarbia from obstructed CO_2_ exchange, hypoxemia with pulse oximetry
Peripheral Muscle Weakness	Fasciculations/weakness in any peripheral muscle group	EMG (weakness, fasciculations), acceleromyography (weakness), laboratories	Impaired neurotransmission, electrolyte abnormalities
	Grip strength decreased	Grip strength meter, neuromuscular monitoring of ulnar nerve, ToF < 80%	-
	Respiratory muscle weakness/hypoventilation	Inspiratory force, spirometry (minute ventilation, respiratory rate)	Hypercarbia, acidosis, hypoxemia
	SIMV etc. to more complete pressure or total ventilatory support	Changes in support requirements	Hypercarbia, acidosis, hypoxemia
	Unassisted breathing on ventilator	Changes in support requirements	Correction of hypercarbia, acidosis, hypoxemia

Increases in serum potassium may be small or large, depending on severity and type of envenoming. Hypercarbia, acidosis and hypoxemia may result form intercostal/diaphragmatic weakness and respiratory fatigue or failure. For example, PaCO_2_ > 50 mmHg, pH < 7.30, PaO_2_ < 70 mmHg suggests inability to clear CO_2_ and require more support than can be provided by SIMV or other pressure support modes. SIMV = synchronized intermittent mandatory ventilation.
